# Comparison of Aggregated N-of-1 Trials with Parallel and Crossover Randomized Controlled Trials Using Simulation Studies

**DOI:** 10.3390/healthcare7040137

**Published:** 2019-11-06

**Authors:** J. Walker Blackston, Andrew G. Chapple, James M. McGree, Suzanne McDonald, Jane Nikles

**Affiliations:** 1 Department of Epidemiology, Tulane University School of Public Health & Tropical Medicine, New Orleans, LA 70112, USA; 2 Biostatistics Program, School of Public Health, Louisiana State University Health Sciences Center, New Orleans, LA 70112, USA; achapp@lsuhsc.edu; 3 School of Mathematical Sciences, Queensland University of Technology, Brisbane 2434, Australia; james.mcgree@qut.edu.au; 4 UQCCR, The University of Queensland, Brisbane 4006, Australia; suzanne.mcdonald@uq.edu.au (S.M.); catherine.nikles@uq.edu.au (J.N.)

**Keywords:** N-of-1 trial, evidence-based medicine, comparative effectiveness, clinical trial, single-case study, simulation study, statistical methods, RCT

## Abstract

*Background:* N-of-1 trials offer an innovative approach to delivering personalized clinical care together with population-level research. While increasingly used, these methods have raised some statistical concerns in the healthcare community. *Methods:* We discuss concerns of selection bias, carryover effects from treatment, and trial data analysis conceptually, then rigorously evaluate concerns of effect sizes, power and sample size through simulation study. Four variance structures for patient heterogeneity and model error are considered in a series of 5000 simulated trials with 3 cycles, which compare aggregated N-of-1 trials to parallel randomized controlled trials (RCTs) and crossover trials. *Results:* Aggregated N-of-1 trials outperformed both traditional parallel RCT and crossover designs when these trial designs were simulated in terms of power and required sample size to obtain a given power. N-of-1 designs resulted in a higher type-I error probability than parallel RCT and cross over designs when moderate-to-strong carryover effects were not considered or in the presence of modeled selection bias. However, N-of-1 designs allowed better estimation of patient-level random effects. These results reinforce the need to account for these factors when planning N-of-1 trials. *Conclusion:* N-of-1 trial designs offer a rigorous method for advancing personalized medicine and healthcare with the potential to minimize costs and resources. Interventions can be tested with adequate power with far fewer patients than traditional RCT and crossover designs. Operating characteristics compare favorably to both traditional RCT and crossover designs.

## 1. Introduction

The N-of-1 trial design is increasingly popular among healthcare researchers and clinicians. N-of-1 trials are increasingly proposed as an alternative to randomized controlled trials (RCTs) [1,2]. N-of-1 trials are particularly useful for evaluating treatment of chronic stable conditions such as attention deficit hyperactivity disorder (ADHD), chronic pain, and many other chronic stable conditions [3,4,5,6,7,8,9]. Such designs can be implemented for a variety of populations (from children to the elderly [10,11,12,13,14]), settings (developed and developing countries [15,16]), demographics (races and ethnicities [17]), and indications (e.g., targeted efforts to deprescribe excessive medication [18]). Taken together, N-of-1 trials could represent the next advance in personalized medicine given their ease of integration with health technology data [19,20].

N-of-1 trials can result in demonstrably higher patient care quality while simultaneously minimizing costs because they identify treatment effectiveness for individual patients [21]. Pooling (aggregating) the effects from a series of N-of-1 trials using standardized protocols has been shown to produce robust effect estimates with fewer individuals required relative to randomized controlled trials [2,12]. Training of investigators in the protocol allows N-of-1 trials to be implemented in the same way at each site and aggregated results to be more accurately generalized. These aggregated N-of-1 trials contain the added benefit of demonstrating clinically meaningful results at reduced sample sizes relative to randomized controlled trials [22] Because they require fewer participants to obtain the same power as an RCT, and thus can achieve the required sample size more quickly, the cost compared to other designs can be less [10] However, their use and appeal vary widely among the healthcare community. Clinicians interviewed by Kravitz et al. [23] to identify clinical issues that manifest as barriers to N-of-1 implementation primarily reported issues with the duration of N-of-1 trials and general lack of procedural knowledge. Work is underway to explore these issues further.

Methodologists have also raised issues with trial design and analysis techniques [12,13,14]. Repeated observations of outcomes across multiple treatment and control periods is required from the participant in an N-of-1 trial, in order to provide sufficient statistical power to detect true treatment effects at the individual level. The need for repeated observations can lead to statistical issues which can be addressed through careful statistical analysis. Selection bias, power, sample size, design and method of data analysis are important considerations for any clinical trial.

### 1.1. Selection Bias, Power, & Sample Size

An issue when conducting aggregated N-of-1 trials (i.e., data aggregated from a series of N-of-1 studies) is the generalizability (external validity) of their results. Selection bias in aggregated N-of-1 trials could result from not including a sufficiently sized, representative sample of participants. This concern is also relevant to RCTs. The external and internal validity of aggregated N-of-1 trials can be limited by three primary forms of selection bias: lack of representativeness due to a small sample size, lack of random sequence allocation of intervention and control periods, and lack of allocation concealment [24]. To address these, one can use block randomization or counterbalancing and single, double, or triple blinding treatment when feasible. Other strategies to increase scientific rigor include repeated assessments within treatment periods, adaptive trial “stopping rules” to terminate trials as soon as negative or positive treatment effects are demonstrated, and applying appropriate design and methods of analysis that yield highest power and effect size without inflating type-I error [23,25]. An important statistical consideration in planning an aggregated N-of-1 study (i.e., data aggregated from a series of N-of-1 studies) involves calculating internally valid power and sample size. Aggregated N-of-1 trials require smaller overall sample sizes than traditional RCTs, as individuals are serving as their own controls. One must account for power needed to achieve identification of individual- and group-level differences resulting from treatment.

### 1.2. Trial Design and Carryover Effects

Carryover effects are present in N-of-1 trials when the effect of a treatment cycle impacts subsequent cycles beyond any assigned washout periods. Washout periods are pre-defined blocks of time in which treated individuals do not receive the treatment of interest to allow time for the effect of a treatment delivered in a previous period to wash out. It is possible that including blocks with varying lengths might best address this issue. Future work might evaluate the number of “control days” could be randomly picked in a set of numbers between 1 and 3 following an “intervention day.” Rather than equal time spent in control and treatment cycles, this randomization of block length potentially addresses the possibility of confounding effects from treatment carryover. Stronger carryover effects may result in more conservative differences in treatment effects due to inflation of type-II error [16]. Thorough reviews of pilot data or relevant literature should be implemented prior to trial design in order to determine the potential length of a carryover effect and inform the design of the N-of-1 study periods [26].

### 1.3. Data Analysis in N-of-1 Trials

There are no known “gold standard” approaches to analyze data for N-of-1 trials. Several approaches have been proposed and are well argued with simulated or trial data [26,27,28]. However, issues of missing data and autocorrelation persist. Many techniques to handle missing data are being developed for both observational and interventional study designs [11,16,17]. Simulation studies can incorporate missingness but are difficult to model similarly to real-world data. Autocorrelation presents an additional complication when analyzing N-of-1 data. Autocorrelation implies a serial dependency within the data collected from the same individual over time—i.e., patient’s stress level today may be highly correlated with their stress yesterday and tomorrow. Thus, it is important to check for autocorrelation in data from N-of-1 trials and address it if detected. Traditional tests of difference such as the *t-*test are not appropriate for analyzing data from N-of-1 trials because they violate key assumptions of statistical independence. Two common methods to address autocorrelation include an autoregressive model or a dynamic model, where autocorrelation is modelled as part of the analysis procedure [23,29]. Random effect parameters can be included to account for autocorrelation in multi-level or hierarchical models.

The aim of this paper is to compare N-of-1 trials to parallel and crossover RCT designs and evaluate (1) effect sizes, (2) power and (3) sample size for each study design through simulation study. Results from this study will provide information about the adequacy and utility of N-of-1 trials compared to traditional trial designs under a range of potential trial conditions.

## 2. Methods

We performed a simulation study to compare the operating characteristics of aggregated N-of-1 trials, RCTs and crossover designs for varying sample sizes and effect sizes. We examined the differences in a proposed trial of a new therapy versus placebo over 3 cycles. These cycles were sampled representatively to include a proportion with cycle effects, without cycle effects and then disproportionally from a population with intentional lack of generalizability. In general, data was generated from:

$$Y_{ick}=\mu_{i}+\tau \;Z_{ick}+\epsilon_{ick},$$

where $Z_{ick}$ is the treatment indicator for patient i  at cycle c=1, 2, 3  and “look” k=1, 2 within a cycle. τ is the treatment effect of interest. We considered $\mu_{i}\sim N\left(\mu ,\;\sigma_{\mu }\right)$ and $\epsilon_{ick}\sim N\left(0,\sigma_{\epsilon }\right)$ and τ=0.25 to be a moderate effect. We looked at 4 different standard deviation structures.

- Scenario 1—Weak heterogeneity and moderate error: $\sigma_{\mu }=0.1$ and $\sigma_{\epsilon }=0.5$.
- Scenario 2—Homogeneity and moderate error: $\sigma_{\mu }=0$ and $\sigma_{\epsilon }=0.5$.
- Scenario 3—Strong heterogeneity and moderate error: $\sigma_{\mu }=0.5$ and $\sigma_{\epsilon }=0.5$.
- Scenario 4—Strong heterogeneity and large error: $\sigma_{\mu }=0.5$ and $\sigma_{\epsilon }=1$.

For each of the four variance settings and each of 5000 simulations, we generated c=3 N-of-1 cycles from the above model. The first cycle’s observations were used for a crossover trial. One of the two observations, including outcome and treatment assignment, in this first cycle were randomly chosen with equal probability for use within the parallel RCT. This ensured that the same data was used in each of the 3 trials to provide a fair comparison. Power was computed as the proportion of times that each simulation rejected the null hypothesis of no treatment effect [30]. This was done using the ‘lmer’ function in R statistical software version 3.5.2, (R Foundation for Statistical Computing, Vienna, Austria), and follows previously proposed approaches to estimate power in N-of-1 studies [30]. Two models were estimated (1) an intercept only model and (2) a model with an intercept and treatment effect, where each fitted model contained patient-specific random effects. The difference in deviance of the two models was then used for the chi-squared test of no treatment effect at the nominal 0.05 type-I error level. Deviance calculations included random effect calculations for each model. *p*-values for this test that were less than 0.05 were considered to be statistically significant. The null treatment effect hypothesis was tested similarly for the crossover design, while a standard linear regression model was used to test this hypothesis for the RCT.

## 3. Results

In each simulation setting, power for N-of-1, parallel RCT and crossover design were computed for maximum sample sizes of n=10, 20, 30, 40, 50, 100, 150, 200  with a fixed treatment effect τ=0.25. After finding the value of n that produced a power above 0.8 for the N-of-1 design, we fixed this sample size and varied τ=0, 0.05, 0.10,…, 1 to determine how each design performed as the true treatment effect increased. Here τ=0 represents a case where the treatment provides no true improvement over placebo, causing rejection of the null hypothesis when it is true, to constitute a type I error.

**Figure 1:**
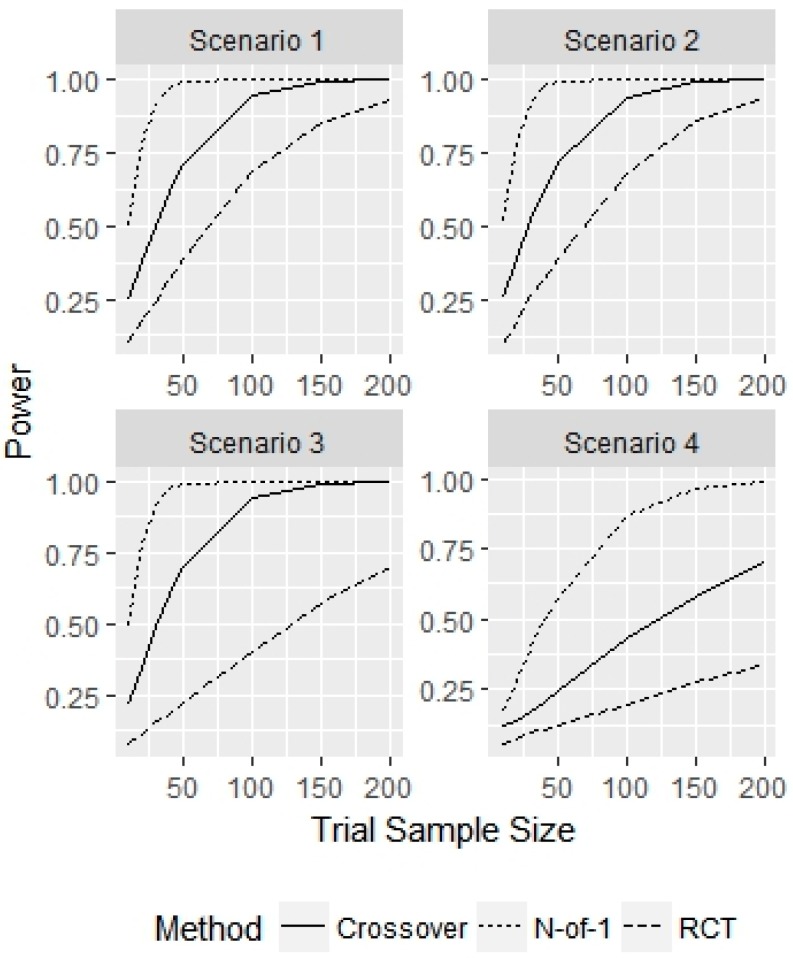
Simulation study for representative sample without washout effects. This figure displays the power for detecting τ=0.25 for given sample sizes and the 3 different designs considered.

First, we examined how N-of-1, parallel RCT and crossover trials performed when all patients come from the population of interest, with true treatment effect τ=0.25, and no washout or carryover effects with a placebo vs treatment comparison in c=3 cycles. Figure 1 displays the power of the 3 designs as a function of trial sample size for the 4 different variance structure scenarios.

Across the 4 scenarios, the N-of-1 sample sizes needed to achieve 80% power with a main effect of τ=0.25 were 30, 30, 30 and 100, respectively. For scenarios 1 and 2, the parallel RCT achieved 80% power with n=150 patients and the crossover design achieved 80% power with n=100 patients. The corresponding power of the N-of-1 designs for these two sample sizes were both 100%. The power of the two alternative designs in scenario 1 at n=30, where the N-of-1 design had a power of 92%, were 32% and 50% for the parallel RCT and crossover designs, respectively. For scenario 2, the power for the N-of-1 design was 92% at n=30, compared to 26% and 52% for the parallel RCT and crossover designs, respectively. In scenario 3, the parallel RCT did not achieve power above 80% for any sample size considered, and the crossover design required 100 patients to obtain a power of 94%, compared to a parallel RCT power of 70% for n=50. For scenario 4, where the random patient effect variance and error variance were largest, neither the parallel or crossover designs achieved power above 80%, with empirical power values of 34% and 70%, respectively for a sample size of n=200. The power for the N-of-1 design for this sample size was 99%.

**Figure 2:**
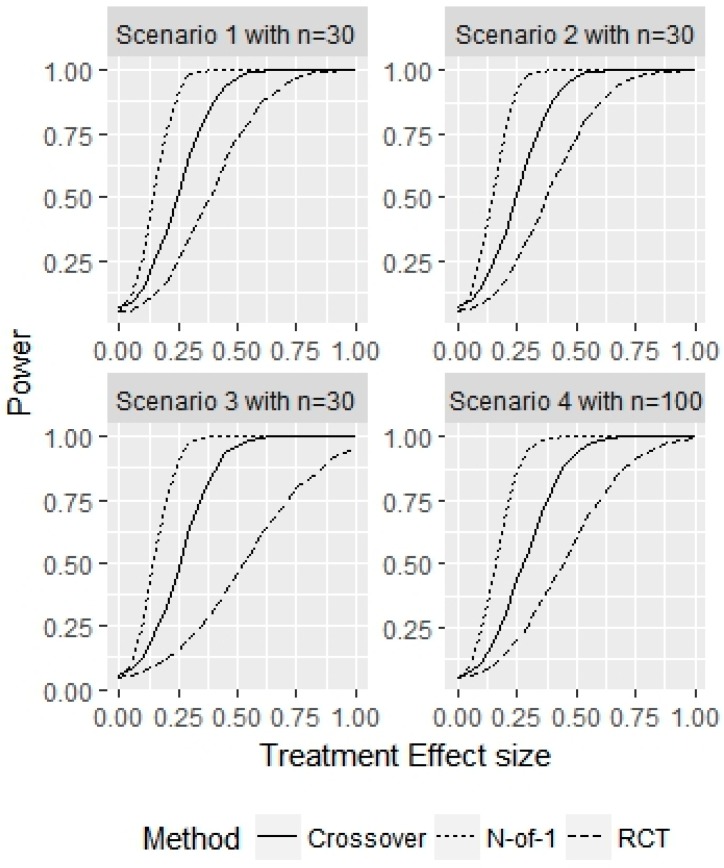
Simulation study for representative sample without washout effects. This figure displays the power for a fixed sample size n with varying true treatment effects τ for the 3 different designs considered. The power for τ=0 represents the type I error probability.

Figure 2 displays the power of a representative sample without washout effects for a fixed sample size in each scenario and a varying treatment effect τ. The sample size used in each scenario corresponded to the minimum sample size needed to achieve at least 80% power for the N-of-1 design. For an effect size of τ=0, the N-of-1 empirical type I error probability was 0.05, 0.05, 0.05, 0.05, for the 4 scenarios, respectively. The empirical type I error probability for the RCT was 0.05, 0.06, 0.04, 0.05 across the 4 scenarios. The empirical type I error probability for the crossover design was 0.07, 0.07, 0.06, 0.05, across the 4 scenarios, respectively. The N-of-1 design best matched the empirical type-I error probability to the desired nominal type I error probability. By design, τ=0.25 produced power figures above 80% for both N-of-1 designs and the considered sample sizes. For parallel RCTs, τ=0.60, 0.50, 0.80, 0.65, and for the crossover designs, τ=0.40, 0.40, 0.40, 0.45 were required to achieve a power of at least 80% across the 4 variance scenarios.

**Figure 3:**
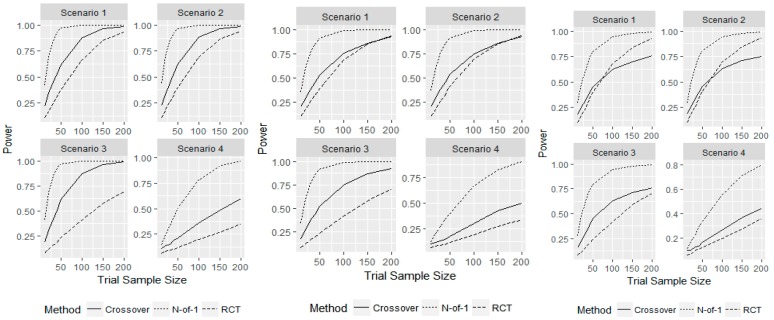
Simulation study for representative sample with a carryover effect size of 0.05 (left), 0.10 (middle) and 0.15 (right). Displays the power for detecting τ=0.25 for given sample sizes and the 3 different designs considered.

Next, we examined the operating characteristics of each design in the presence of carryover effects. We increased patient outcomes by 0.05, 0.1, and 0.15 if they had just received the new therapy. By increasing patient outcomes in this manner, we do not consider length of cycle or washout, and remove the time-washout relationship from this simulation study. These represented small, medium, and large carryover effects compared to the true treatment effect of 0.25. Thus, patient outcomes could be increased for placebo or therapy outcomes within a cycle, or during the next cycle in N-of-1 designs. For example, if a patient received treatment in the first period in a cycle, their next outcome within the cycle for placebo will be increased due to the washout effect. Similarly, if a treatment is given at the end of a cycle, the next cycles first outcome – whether from a patient receiving treatment or placebo—will be increased from the washout effect. This did not affect parallel RCT operating characteristics as patients only receive either the placebo or treatment and only have one observation. For the crossover design, carryover effects were only seen if a patient received the new therapy before the placebo treatment, whereas carryover effects could be seen within each cycle and in between cycles in N-of-1 studies. Figure 3 displays the power for sample sizes of n=10, 20, 30, 40, 50, 100, 150, 200 and a fixed treatment effect τ=0.25 for the three different carryover effects considered.

The sample sizes required for the N-of-1 design to achieve at least 80% power were increased to  n=40, 40, 40, 150, respectively, for a moderate carryover effect size of 0.1. The power of the crossover design at these sample sizes was 45%, 45%, 43%, and 43%, which was still higher than the parallel RCT despite it having no change in operating characteristics from the carryover effect. The N-of-1 designs required a sample size of n=30, 30, 30, 150 to achieve 80% power with a carryover effect of 0.05 and n=50, 50, 100, 200 for a carryover effect of 0.15. The N-of-1 designs achieved an exact power of 80% for n=200 and a large carryover effect of 0.15. The power of the crossover design for the same required sample sizes for 80% power for N-of-1 design with small and large carryover effects were 45%, 45%, 42%, 48%, and 45%, 46%, 63%, and 44%, across the four scenarios, respectively. When a true treatment effect was present, the N-of-1 trial designs had a higher power to detect a difference than traditional parallel RCT and crossover designs for any carryover effect size, if this effect was not larger than the treatment effect. For a very large carryover effect of 0.15, the power curves for the parallel RCT and crossover designs crossed, indicating that with a large enough sample size the parallel RCT outperformed the crossover design. This is likely due to crossover designs only having a carryover effect when the placebo is given after the treatment, which decreases the chances of detecting a true treatment effect.

**Figure 4:**
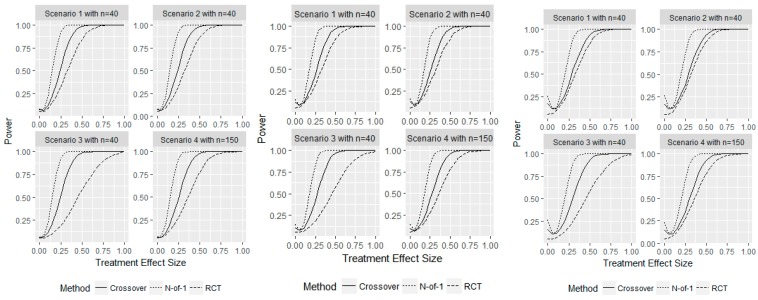
Simulation study for representative sample with a washout effect size of 0.05 (left), 0.10 (middle), and 0.15 (right). Displays the power for a fixed sample size n with varying true treatment effects τ for the 3 different designs considered. The power for τ=0 represents the type I error probability.

Next, we examined the operating characteristics for varying τ at each sample size required to achieve 80% power in the N-of-1 design for the small, moderate, and large carryover effect sizes of 0.05, 0.10, and 0.15. This also allowed us to examine the design performance as the treatment effect changes relative to the carryover effect. First, we should note that the empirical probability of a type I error was inflated for both the N-of-1 designs and crossover designs compared to the parallel RCT. The N-of-1 design resulted in empirical type I error probabilities of 15%, 15%, 14%, 14%, and the crossover design had empirical type I error probabilities of 11%, 12%, 11%, and 9% for a moderate effect size of 0.1. For a small carryover effect of 0.05, the N-of-1 and crossover designs had better controlled type I error probabilities of 8%, 7%, 6% 7%, and 7%, 7%, 6%, 6%, respectively. For a large carryover effect of 0.15, which is over half the true treatment effect size, the type I errors across the 4 scenarios considered were 25%, 26%, 27%, 23% for the N-of-1 design and 17%, 17%, 16%, 15% for the crossover design. These inflations are entirely due to the carryover effect and can be seen in the curved upwards left tail of the 4 plots in Figure 4. For the crossover design, if a patient receives the placebo after treatment, the estimated treatment effect will be negative when τ=0. Likewise, the N-of-1 design can have treatment effect estimation bias for the placebo within a cycle if the new therapy is given first and biased for the new therapy if it is given at the end of a cycle and beginning of the following cycle, without washout. Additionally, we fit an N-of-1 model that adjusted for carryover effects by including an additional binary fixed effect corresponding to whether or not the patient received treatment in the previous treatment period. The type I error and corresponding power for different values of τ were nearly identical when controlling for carryover effects. These results indicate that special care should be given to controlling for carryover effects, particularly to avoid type I error. Still, when τ>0, the power is higher for the N-of-1 design for any effect size, with the crossover design achieving at least 80% power with effect sizes of τ=0.40 for each scenario. Normally data from days when there could be a carryover effect is not used in the analysis.

Finally, we examined the extent of issues that N-of-1 trials can have when there is a non-representative sample for the population of interest (i.e., selection bias). We have already demonstrated that if samples are drawn from the population of interest, N-of-1 trials obtain desired power with fewer patients than parallel RCTs or crossover designs and maintain type I error constraints when carryover effects are not present.

Consider an N-of-1 trial of a new therapy versus placebo targeting improvements of psychological health in adults age 25–50 years old. It is plausible that our population contains individuals with disproportionately high or low baseline risk of poor psychological health relative to our target population. If we sample from this sub-population our statistical conclusions about the population of interest may be incorrect.

**Figure 5:**
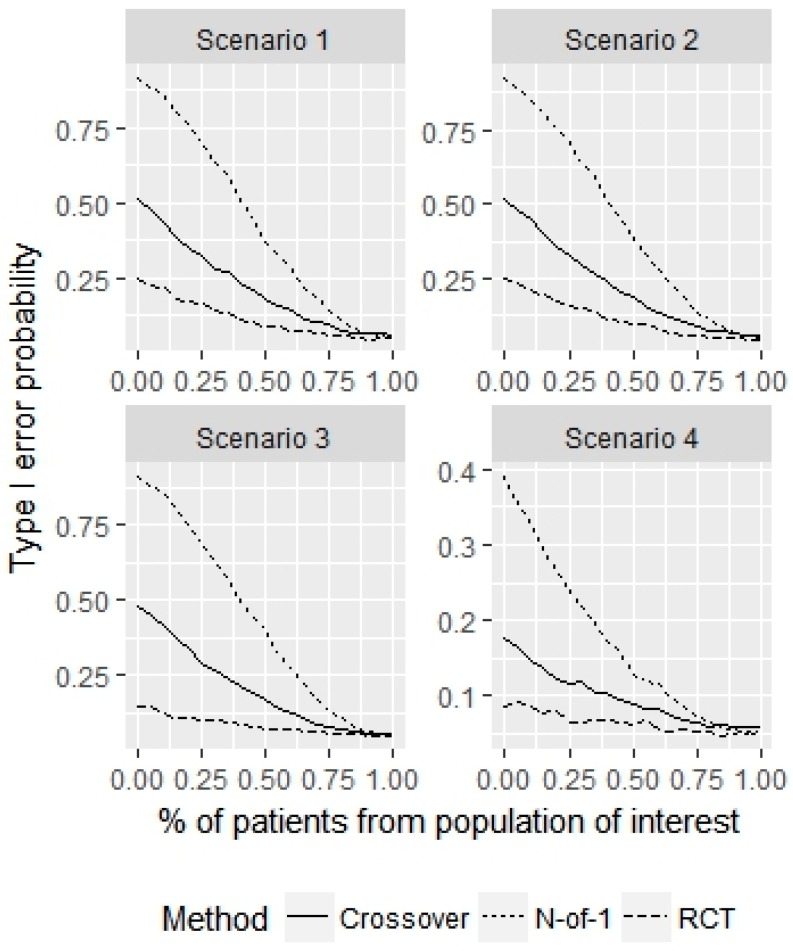
Simulation study for non-representative sample of size 30. This figure displays the probability of making a false discovery (i.e., type I error) for the population as a function of misrepresentation proportions.

To test performance under selection bias, we performed the following simulation experiment using the 4 error variance structures described above. With probability p, we sample patients for our N-of-1 trial from the population of interest, which has no treatment advantage (τ=0). With probability 1−p, we sample patients from a sub-population that has a treatment effect of τ=0.25. The probability that we falsely conclude that a treatment effect is present in the population of interest (i.e., make a type I error) is plotted in Figure 5 for varying p and an N-of-1 sample size of 30.

When p=1, the type I error is 0.05, as desired for each of the 3 designs in each scenario. But when p→0, the empirical probability of type I error increases, particularly for the N-of-1 design. When p=0.7, indicating that we incorrectly sample on average 9 patients from the sub-population, the type I error for the parallel RCT, crossover and N-of-1 designs are (7%, 6%, 6%, 6%), (11%, 11%, 9%, 7%), (19%, 19%, 17%, 9%), across the 4 scenarios, respectively.

When p=0.5, indicating that we incorrectly sample on average 15 patients from the sub-population, the type I error for the parallel RCT, crossover and N-of-1 designs are (9%, 10%, 7%, 6%), (18%, 19%, 17%, 9%), (37%, 39%, 40%, 13%), across the 4 scenarios, respectively. This indicates that special care must be taken to ensure that the sample represents the population of interest for the crossover design and especially for the N-of-1 design. A greater number of observations from the same patient compounds the error caused by non-representative sampling.

**Figure 6:**
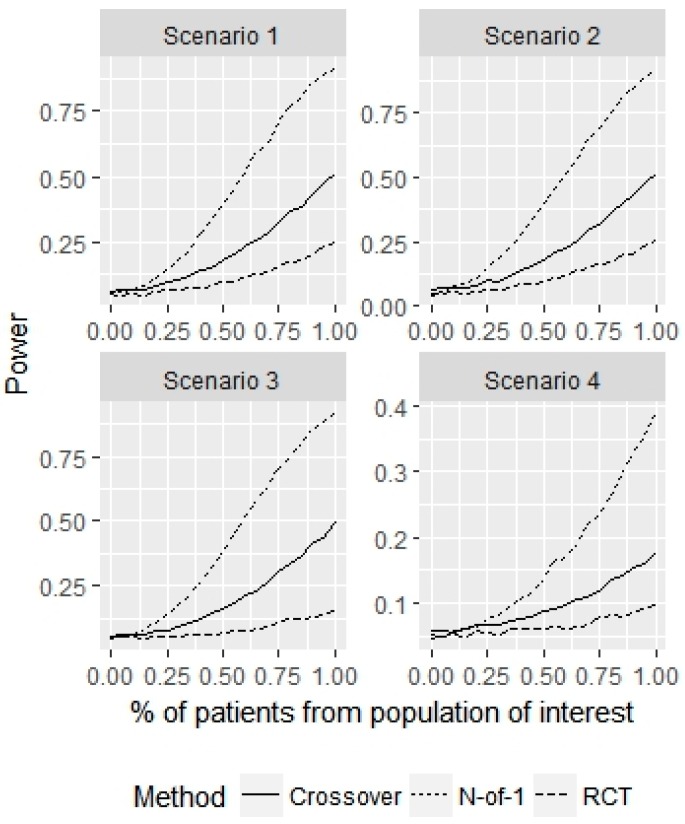
Simulation study for non-representative sample of size 30. This figure displays the power for the population of interest as a function of the proportion of patients sampled from this population.

Next, we examined the power in a similar manner, when the population of interest truly has a treatment effect of τ=0.25 and patients from some sub-population have a true treatment effect of τ=0 (i.e., the treatment does not work for the sub-population). We examine the power for varying p and a fixed sample size of 30 in Figure 6.

We see that for all 4 scenarios, the power increases dramatically as p increases. It is essential that patients represent the population of interest to generalize conclusions from an N-of-1 trial. If p=0.8, indicating that about six patients are sampled from the non-representative population, the power for the N-of-1 design is (81%, 81%, 80%, 30%) for the 4 scenarios, compared to (18%, 17%, 12%, 8%) and (37%, 36%, 34%, 14%) for the parallel RCT and crossover designs, respectively. This indicates that the non-representative sampling from a sub-population not of interest has a much greater effect on type I error probability than power for the N-of-1 design compared to the parallel RCT and crossover designs.

**Figure 7:**
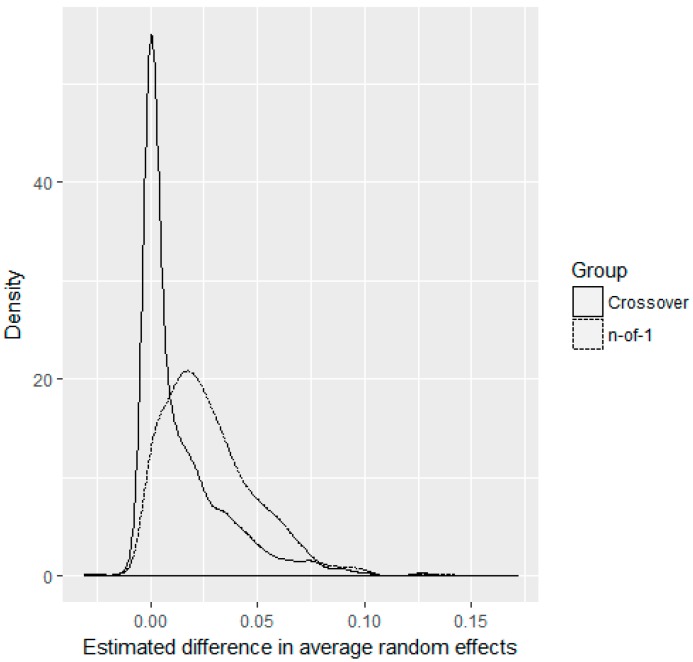
Average estimated random effect differences between non-representative and representative patients. Densities are shown for scenario 1 with p=0.5, n=100, and 1000 simulations for N-of-1 and crossover designs.

In practice, we will never know if patients enrolled in a trial are representative of our treatment population or not. While N-of-1 designs exacerbate this problem compared to parallel RCT and crossover designs through leveraging multiple observations on each patient under two different treatments, N-of-1 designs also allow better estimation of patient-level random effects, which might possibly be used in future methodological improvements to better determine which patients are representative of the target population. As an example, to show this we simulated 1000 trials from scenario 1, with n=100 and p=0.5, i.e., about 50% of patients enrolled in the trial have no treatment effect, whereas the other 50% have a treatment effect of 0.25. In each simulation, we computed the average random effect for representative and non-representative patients using both the N-of-1 and crossover designs. Parallel RCTs cannot estimate patient-level random effects because they only have one observation on each patient. Figure 7 displays the density of the differences in average random effects between the non-representative and representative patients for both the crossover and N-of-1 designs. We see that the N-of-1 design correctly identified that the individual random effects of the non-representative group are higher than for our population of interest, as indicated by the shift in densities. These results were similar, but less striking for n=30, and more apparent with more cycles in the N-of-1 design. While these are individual random effects, and not individual treatment effects, these results suggest that future methodological advancements may be able to cluster patient treatment effects to determine existence of subgroups within patient cohorts.

## 4. Discussion

N-of-1 trial designs offer a rigorous method for advancing personalized medicine and healthcare while minimizing costs and resources, provided treatments are ethically designed for chronic stable conditions with sufficient washout periods. We performed a simulation study to examine the operating characteristics of N-of-1 designs compared to parallel RCT and crossover designs. Our results follow closely from previous theoretical results which demonstrated simulated sample sizes required to achieve a given power were lower for N-of-1 trials than for parallel RCTs, as the number of cycles administered increased [31]. We validated this theoretical result through simulations and showed that N-of-1 operating characteristics were superior to those of crossover designs. We also examined the effects of carryover and sample representativeness on the operating characteristics of each of the three trial designs. Carryover effects can result in inflated type I error probabilities for both crossover and especially N-of-1 designs, but when treatment effects are present, N-of-1 designs have better power than both crossover and parallel RCT designs, regardless of carryover effect size. When trial samples are not fully representative of the target population, all three trial designs make incorrect conclusions about the treatment effects in the target population, but N-of-1 designs make incorrect conclusions far more often due to increased numbers of observations on each patient. However, due to multiple observations taken on each patient under different treatment periods, N-of-1 designs provide the opportunity to identify patients who are not alike within the population of interest, suggesting the need for Bayesian clustering methods to be further developed and implemented [12,23,26,32].

In addition to expanding the analytical approaches to quantifying carryover effects (i.e., slow vs. long), N-of-1 studies offer a unique advantage: they can often provide their own “pilot data” through initial phases prior to data aggregation or meta-analysis. Researchers with little prior information on expected carryover effects for a phenomenon could gather pilot data before conducting a full trial.

We have shown that N-of-1 trials have more power at lower sample sizes compared to parallel RCT and crossover designs. This finding has also been shown in similar studies [31,33]. N-of-1 studies should be considered high-grade evidence for informing subsequent research, e.g., proof-of-concept, or traditional randomized control trial designs. We posit that N-of-1 trials offer benefits not present in traditional designs.

Limitations

We acknowledge several limitations. First, testing the full range of possible design variants would exceed the scope of this paper. Some designs were arbitrary and unlikely to occur in true trial conditions such as scenarios with very high or low variance or sample sizes in the 10’s or 200’s. These structures are necessary to consider, however, as they demonstrate the comparative effects on each trial design when scaling up or down from average sample sizes. Thus, attempts to cover this range substantially, but also efficiently, were emphasized. Indeed, not all statistical challenges present with N-of-1s could be evaluated with equal rigor—selection bias cannot be modelled as comprehensively or accurately as power, sample size, or even carryover effects. Subsequent research is warranted which might further model and formalize selection bias hierarchically with other concerns.

## 5. Conclusions

N-of-1 trials can be demonstrably effective in ascertaining treatment effects, both at individual and population levels. Interventions can be tested with adequate power with far fewer patients than traditional RCT and crossover designs. Operating characteristics compare favorably to both traditional RCT and crossover designs. 

N-of-1 trials demonstrate potential to significantly improve clinical decision-making. Physicians can continue to treat individuals where improvement is demonstrable and stop treatment for those who have harmful or null effects. Such trial designs may offer a vital *complement* to traditional research methods. Our findings provide further evidence that N-of-1 trials can produce rigorous, evidence-based results to inform personalized healthcare.
